# Albumin Protein Impact on Early-Stage *In Vitro* Biodegradation of Magnesium Alloy (WE43)

**DOI:** 10.1021/acsami.3c12381

**Published:** 2023-12-18

**Authors:** Amin Imani, Ehsan Rahimi, Maria Lekka, Francesco Andreatta, Michele Magnan, Yaiza Gonzalez-Garcia, Arjan Mol, R. K. Singh Raman, Lorenzo Fedrizzi, Edouard Asselin

**Affiliations:** † Department of Materials Engineering, The University of British Columbia, Vancouver, BC V6T 1Z4, Canada; ‡ Department of Mechanical and Aerospace Engineering, Monash University, Clayton, VIC 3800, Australia; § Department of Materials Science and Engineering, Delft University of Technology, Mekelweg 2, 2628 CD Delft, The Netherlands; ∥ CIDETEC, Basque Research and Technology Alliance (BRTA), 20014 Donostia, San Sebastián, Spain; ⊥ Polytechnic Department of Engineering and Architecture, University of Udine, 33100 Udine, Italy; # Department of Chemical and Biological Engineering, Monash University, Clayton, VIC 3800, Australia

**Keywords:** magnesium alloy, protein adsorption, corrosion, biodegradation, surface potential

## Abstract

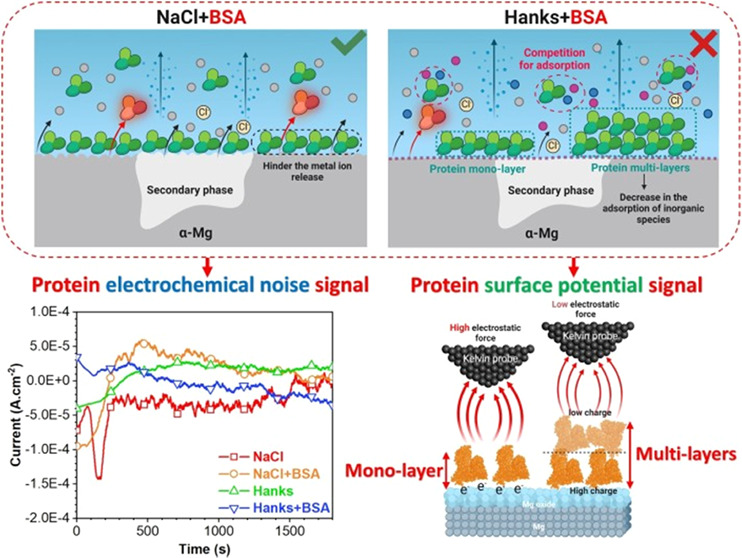

Mg and its alloys are promising biodegradable materials for orthopedic implants and cardiovascular stents. The first interactions of protein molecules with Mg alloy surfaces have a substantial impact on their biocompatibility and biodegradation. We investigate the early-stage electrochemical, chemical, morphological, and electrical surface potential changes of alloy WE43 in either 154 mM NaCl or Hanks’ simulated physiological solutions in the absence or presence of bovine serum albumin (BSA) protein. WE43 had the lowest electrochemical current noise (ECN) fluctuations, the highest noise resistance (*Z*_n_ = 1774 Ω·cm^2^), and the highest total impedance (|*Z*| = 332 Ω·cm^2^) when immersed for 30 min in Hanks’ solution. The highest ECN, lowest *Z*_n_ (1430 Ω·cm^2^), and |Z| (49 Ω·cm^2^) were observed in the NaCl solution. In the solutions containing BSA, a unique dual-mode biodegradation was observed. Adding BSA to a NaCl solution increased |*Z*| from 49 to 97 Ω·cm^2^ and decreased the ECN signal of the alloy, i.e., the BSA inhibited corrosion. On the other hand, the presence of BSA in Hanks’ solution increased the rate of biodegradation by decreasing both *Z*_n_ and |*Z*| while increasing ECN. Finally, using scanning Kelvin probe force microscopy (SKPFM), we observed an adsorbed nanolayer of BSA with aggregated and fibrillar morphology only in Hanks’ solution, where the electrical surface potential was 52 mV lower than that of the Mg oxide layer.

## 1 Introduction

The unique benefits of certain Mg alloys, such as their biodegradability, reasonable mechanical properties similar to bone tissue, and nontoxicity, have prompted researchers to focus on improving their in-service properties, particularly their long-term durability.^1−4^ Mg alloys have numerous medical applications, including as temporary non-load-bearing bone implants^5^ or bone fixations,^6,7^ scaffolds for tissue engineering,^8,9^ and cardiovascular stents.^10^ However, the biodegradation resistance of Mg alloys remains low, especially in human physiological media containing various ions, proteins, cells, and inflammatory agents.^11,12^ Inorganic ions (e.g., Cl^–^, H_2_PO_4_^–^, HPO_4_^2–^, Ca^2+^, HCO_3_^–^) and protein molecules (albumin, fibronectin, etc.) can reduce or accelerate the rate of deterioration of Mg alloys^2,13,14^ depending on a variety of factors that include ion type, protein concentration, alloy microstructure, alloy chemical composition, and exposure time.^15^

Depending on their molecular structure,^2^ adsorbed proteins, either as single molecules or as nanofilms (e.g., mono- or multilayer), are frequently considered to be electrically conductive soft matter. Consequently, the specific electrical conductivity (EC) of adsorbed protein nanofilms on biomedical surfaces can significantly influence subsequent biological events, particularly electrochemical interactions at oxide/protein/electrolyte interfaces.^6^ It is known that protein adsorption on biomaterial surfaces is a complex process that involves electrostatic, hydrophobic, van der Waals, and hydrogen-bonding interactions.^16^ Protein molecules are capable of instantly adsorbing onto biomaterial surfaces, which can initiate the formation of a biofilm, followed by rearrangement, biodegradation, displacement (Vroman effect), or detachment, leading to the formation of protein-metal complexes/conjugates.^6,8^ However, these protein-adsorption-related biodegradation mechanisms are complicated in the case of biodegradable or bioactive surfaces, such as Mg and Mg alloys. According to a review,^10^ the action of bovine serum albumin (BSA) is time-dependent, initially inhibiting the corrosion processes of Mg alloys, followed by an acceleration of metal dissolution after prolonged immersion. Similarly, within 4000 s of immersion in a physiological solution, the biodegradation rate of Mg alloys was observed to decrease with increasing concentration of BSA protein.^17^ Other research using molecular dynamics simulations revealed that fibronectin molecules have a lower tendency to adsorb on the secondary phases than on the α-Mg (matrix) due to their higher water layer content, lower number of anchored residues, and weaker interaction strength.^11^

The improved biological properties of Mg-based alloys are strongly related to enhanced corrosion resistance.^18^ Biological cells are very sensitive to environmental fluctuations, and the corrosion of Mg-based alloys may lead to the formation of metal ions, hydrogen bubbles, and an alkaline environment.^19^ This, in turn, may have cytotoxic effects on biological cells and reduce biocompatibility. Prior research on in vitro degradation and biocompatibility of WE43, ZK60, and AZ91 alloys showed that these alloys do not induce cytotoxicity.^20^ However, it was mentioned that excessively high concentrations of Mg and Al ions in the culture medium caused increased levels of cellular DNA damage. In general, Mg-based alloys exhibit good antibacterial activity that can fight bacterial proliferation, adhesion, and biofilm formation.^21−23^ The bactericidal effect of Mg-based alloys during the degradation process is attributed to several factors, such as the concentration of Mg^2+^ ions, increased alkalinity, and released magnesium-based nanoparticles, such as magnesium oxide and magnesium hydroxide particles.^24−26^

In the context of WE43 alloys, yttrium plays a critical role in providing enhanced corrosion resistance through multiple mechanisms. It can stabilize the alloy’s microstructure, forming stable compounds with magnesium (e.g., Mg_14_Nd_2_Y) that enhance resistance to corrosion.^27−29^ Additionally, it facilitates the formation of a protective layer (Y_2_O_3_ and Y(OH)_3_) on the alloy’s surface, serving as a barrier against corrosive agents.^29,30^ Yttrium improves resistance to crevice corrosion and contributes to the alloy’s mechanical strength, reducing susceptibility to localized corrosion.

The investigation of protein-adsorption-related biodegradation mechanisms on biodegradable or bioactive surfaces, such as Mg alloys, remains a complex and crucial area of research. Previous studies^10,31^ have shown that protein molecules, including albumin, can significantly influence the corrosion processes and biodegradation rate of Mg alloys. The concentration of BSA was observed to influence the biodegradation rate of Mg alloys.^32,33^ On the other hand, when investigating the action of BSA, it was found to initially inhibit corrosion processes, followed by an acceleration of metal dissolution after prolonged immersion.^15,16^ Studying the early stage of Mg degradation in simulated body fluids is crucial for understanding its initial response, biocompatibility, corrosion rate, optimization, safety, and eventual clinical use.^32,34^ However, the specific role of BSA protein in the early-stage (up to 1 h) biodegradation mechanism of magnesium alloys, particularly WE43 alloy, in simple and complex simulated physiological media, such as 154 mM NaCl and Hanks’ solutions, remains largely unexplored. Therefore, this study aims to fill this research gap by elucidating BSA’s impact on the biodegradation behavior of WE43 using advanced techniques. Through the use of electrochemical noise (EN), X-ray photoelectron spectroscopy (XPS), atomic force microscopy (AFM), and scanning Kelvin probe force microscopy (SKPFM), we aim to visualize the electrochemical response, chemical composition, morphological, and electrical surface potential of the adsorbed protein nanofilm on the complex oxide layer of the magnesium alloy. This detailed visualization of the protein nanofilm’s interaction can lead to actionable outcomes such as the development of more biocompatible implants, improved corrosion mitigation strategies, and the design of novel materials with tailored surface properties for specific applications.

## 2 Experimental Procedure

### 2.1 Materials

As-cast WE43-(T5) Mg alloy was supplied by Xi’an Yuechen Metal Products Co. Ltd. (Shaanxi, China). We cut specimens with a thickness of 5 mm and a surface area of 1 cm^2^ from a bar of the WE43 alloy. After a multiacid digestion (HCl, HNO_3_, and HF in a molar ratio of 30:10:1), the chemical composition (atom %) of the WE43 alloy (91.67 Mg, 3.87 Y, 2.18 Nd, 0.91 Zr, and 1.37 RE) was determined using inductively coupled plasma-optical emission spectroscopy (ICP-OES). Before the electrochemical tests, specimens were sequentially polished up to 2500 grit in aqueous solution. The direction of the polishing was changed three times by a 90° specimen rotation to ensure uniform polishing. To achieve a mirror-like surface finish, the samples were further polished with 0.02 μm silica dispersed in ethanol. Subsequently, the specimens were washed with ethanol, ultrasonically treated in acetone for 20 min, and dried in a stream of air.

### 2.2 Electrolyte and Electrochemical Measurements

The electrochemical response of WE43 was studied in simulated body fluids, including 0.154 M NaCl (0.9 wt %) or Hanks’ (according to H8264 (without glucose), Sigma-Aldrich) solutions containing 4 g L^–1^ of BSA protein (lyophilized powder; 96% agarose gel electrophoresis, Sigma-Aldrich) at pH 7.4 ± 1, 37 ± 1 °C. The electrochemical measurements were performed using an AUTOLAB PGSTAT302 potentiostat in a conventional three-electrode electrochemical cell with a Ag/AgCl/KCl_sat_ (+219 mV vs SHE) reference electrode, a Pt wire counter electrode, and the WE43 specimen as the working electrode. After 30 min of immersion in various environments, electrochemical impedance spectroscopy (EIS) measurements were conducted in the frequency range of 100 kHz to 10 mHz, using a sinusoidal excitation signal of 10 mV at open circuit potential (OCP) conditions. In the electrochemical noise (EN) technique, the electrochemical current and potential noise (ECN and EPN) were recorded simultaneously by electrically connecting two identical (e.g., same surface area and shape) WE43 working electrodes and an Ag/AgCl/KCl_sat_ reference electrode under open circuit conditions. The EN measurements were performed over a period of 1800 at 0.2 s intervals, resulting in a frequency range of approximately 0.5 mHz to 2.5 Hz as determined by the following equations: *f*_max_ = 1/2Δ*t* and *f*_min_ = 1/NΔt, where *t* and *N* represent the sample interval and the total number of data records, respectively. Due to the presence of a complex and heterogeneous system, the power spectral density (PSD) of the EPN and ECN as well as the noise resistance (*Z*_n_) were analyzed because the EN fluctuations were not simple signals related to the relative complexity of the overall system studied: various Mg phases, inorganic and organic species in solution such as phosphates, calcium, protein molecules, etc. All our experiments were performed in triplicate, and the representative data from these replicates were presented in the article.

The PSD is a type of spectrum that characterizes the frequency content of a random signal or the distribution of the signal’s power in the frequency domain.^35−37^ The Fast Fourier Transform (FFT) algorithm is the most frequently used method to model the PSD of a random signal. However, the maximum entropy method (MEM) was developed as an alternative. The MEM is supposedly superior to the FFT for corrosion studies in the following ways: (a) it only requires a single time record for computation, (b) it is significantly quicker than the FFT method, (c) it produces a smoother spectrum than the FFT method, and (d) it permits computation at frequencies lower than the inverse of the acquisition time. The mathematical discussion of the PSD and its autocorrelation functions is available elsewhere.^38,39^ The PSD analyses of EPN, ECN, and *Z*_n_ were computed by using the Hanning windows function for FFT and square windows within the MEM.

### 2.3 Surface Characterization by SEM-EDXS and AFM/SKPFM

The topography and electrical surface potential evolution of WE43 were visualized using a combination of SEM, AFM, and SKPFM surface analyses. The microscopy observations were conducted on as-polished (control) and 10 min immersed samples in different simulated body solutions (NaCl and Hanks) with or without BSA protein. The field emission-scanning electron microscopy (FE)-SEM instrument was a JSM-7610FPlus device (JEOL) energy-dispersive X-ray spectrometer (EDXS), Oxford X-MAX20. All SEM maps were acquired at a working distance of 15 mm with an accelerating voltage of 5 kV, and in secondary electron (SE) mode. A Nanoscope IIIa Multimode device with an n-type doped silicon pyramid single-crystal tip coated with PtIr5 (SCM-Pit probe, tip radius, and height were 20 nm and 10–15 μm, respectively) was used to perform the AFM and SKPFM surface analyses. Surface potential images were captured in dual scan mode. Using the tapping mode, surface topography maps were recorded during the initial scan. The tip was then raised to 100 nm, and the surface potential signal was recorded by following the topography contour from the initial scan. All topographic and surface potential maps were acquired with a scan frequency rate of 0.2 Hz, a pixel resolution of 512 × 512, zero-bias voltage, at 27 °C in 28% relative humidity air atmosphere. The histogram and PDS analyses of topography and surface potential distribution were carried out in accordance with the methodolog y used in ref^16^^16^.

### 2.4 Chemical Surface Characterization by XPS

The chemical composition of the WE43 surface film (both the inorganic and organic components) was measured using a Kratos Analytical Axis ULTRA spectrometer containing a DLD spectrometer using a monochromatic aluminum source (AlKα, 1486.6 eV) operating at 150 W (10 mA emission current and 15 kV HT). Analysis was carried out on a 700 × 300 μm^2^ area of the sample. Survey scans were obtained at a 1 eV step size and pass energy of 160 eV, and averaged over two scans using Vision Processing software by Kratos Analytical. The kinetic energy of the photoelectrons was measured at a 90° takeoff, and the vacuum in the analysis chamber was approximately 5 × 10^–10^ Torr.

### 2.5 Mg^2+^ and H_2_ Release

To determine the concentration of released Mg^2+^ ions as a function of immersion time, WE43 samples were immersed in NaCl and Hanks’ solution (at 37 ± 0.5 °C), and the Mg^2+^ concentration was analyzed using a Hanna Instruments HI97752 portable photometer. The hydrogen evolution rate (HER) at 37 ± 0.5 °C was measured based on the method described by Song et al.^40^ Briefly, a known volume of the test solution was added to a beaker to cover the sample surface. To collect the H_2_ gas being released from the alloy surface, an inverted funnel and a graduated buret were placed over the sample. By measuring the electrolyte level in the buret, the volume of hydrogen gas was calculated.

## 3 Results and Discussion

### 3.1 Microstructural and Surface Potential Analysis of WE43

**Figure 1:**
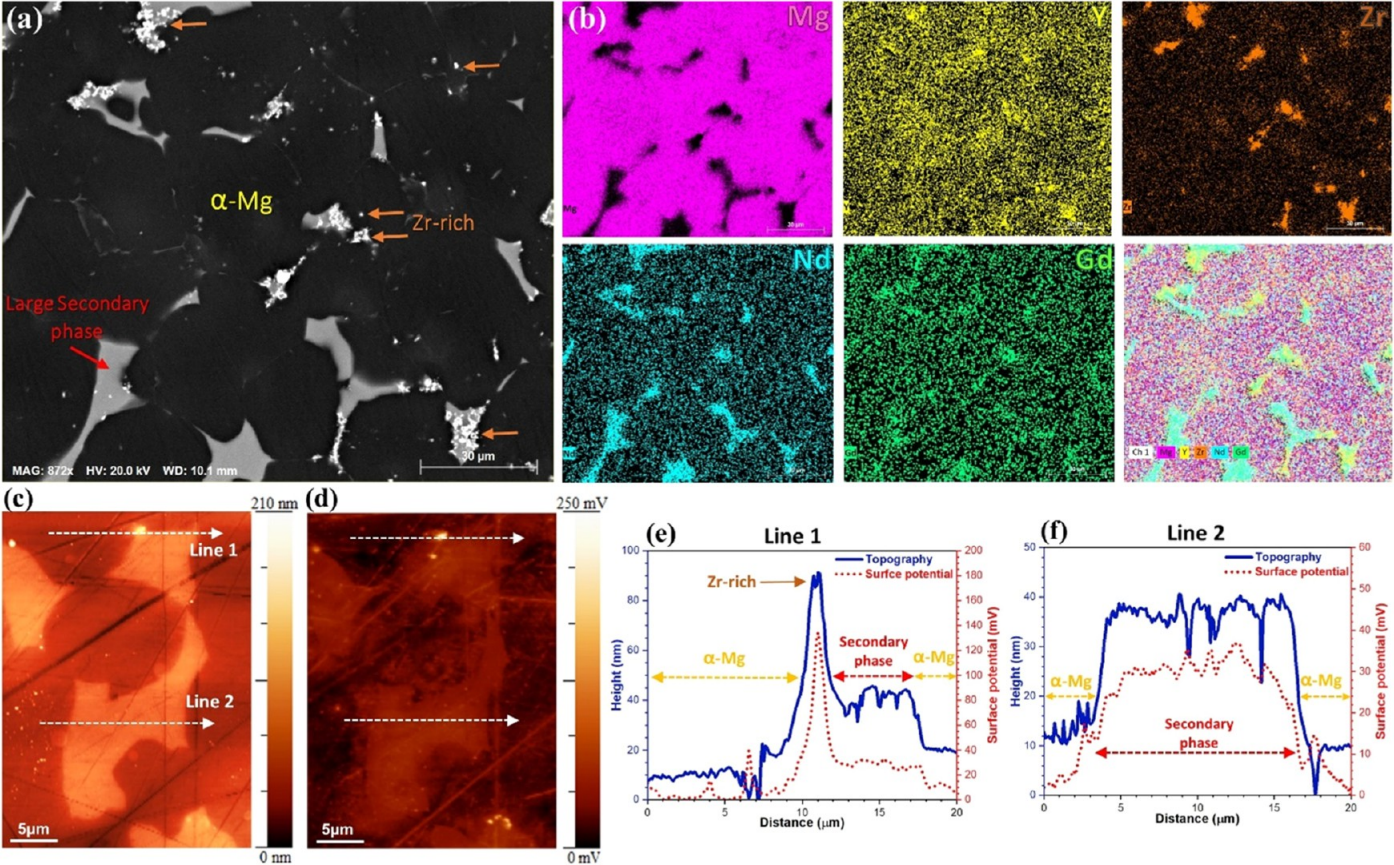
(a) SEM image, (b) EDXS elemental maps, (c) AFM, and (d) SKPFM surface potential maps of as-polished WE 43. (e, f) Topography and surface potential line profiles along the lines seen in (c) and (d).

Figure 1a,b shows an SEM image and corresponding EDXS elemental maps for WE43, revealing its distinct three-region structure: the α-Mg (matrix), a large secondary phase (LSP), and a Zr-rich phase (the chemical composition of the individual phases is shown in Table 1). The LSPs, which contain a higher concentration of Nd and Gd, are spread out randomly around the edges of the matrix grains. The Zr-rich phases mostly form at the boundary between the LSP and matrix, and they are only sparsely distributed in α-Mg that are consistent with the reported literature.^15,16,41^ As can be seen in the EDXS maps, Y is primarily precipitated in Zr-rich phases and is more evenly distributed around the LSPs than in their bulk region.

**Table 1:** Chemical Composition of the Different Individual Phases in WE43

	chemical composition (atom %)				
phase	Mg	Y	Nd	Zr	Gd
α-Mg	96.77	1.43	0.49	1.04	0.27
LSP	89.08	1.61	5.78	1.67	1.86
Zr-rich	74.16	6.13	2.64	15.83	1.24

In a further analysis using AFM/SKPFM, detailed information about the topography and surface potential of different regions in the sample, namely, the α-Mg, LSP, and Zr-rich regions, was obtained. These findings are presented visually in Figure 1c,d, where the topography and surface potential maps are displayed. The surface potential of a material is closely related to its electronic properties, particularly the strength of the electrical surface potential signal, which is directly correlated with the material’s work function energy (WFE).^16^ The distribution of elements within and between phases can impact the electrical surface potential and subsequently the WFE. This non-uniform distribution of elements within the sample can influence the material’s corrosion behavior.

By examining the SKPFM map in Figure 1d, it is evident that the LSP phases exhibit a higher electrical surface potential compared to the α-Mg (matrix). This observation holds true even when considering the negligible influence of differences in surface roughness on the surface potential values.^42,43^ Furthermore, SEM/EDXS maps confirm the presence of small-sized, bright spots with the highest surface potential, which align with the Zr-rich regions.

Importantly, each phase within the alloy possesses distinctive electrical surface potentials, or WFEs. These unique properties influence the tendency of valence electrons to transfer and participate in electrochemical reactions at the metal/electrolyte interface. Consequently, the presence of different electrical surface potentials or WFEs at various interfaces in this alloy can create a microgalvanic driving force, leading to localized corrosion. This prediction is supported by the surface potential line profiles in Figure 1e,f. It should be noted that the corrosion behavior of a material is highly sensitive to environmental factors such as pH levels, the presence of various ions, and other variables. Additionally, the kinetics of the electrochemical reactions involved in corrosion play a significant role. By comprehending these essential elements, one can reveal the underlying reasons behind the susceptibility of Mg-based alloys to corrosion and understand the influence of protein nanofilms on the degradation process.

It is apparent that there is no universal correlation between the contrasts measured by SKPFM and subsequent corrosion behavior, despite the presence of such a correlation in many cases.^44^ Therefore, it is critical to recognize that the relationship between surface potential and local electrochemistry is relevant, but not straightforward.^45^ As a result, it is essential to exercise caution and supplement surface potential data with electrochemical data as well as information on composition and corrosion morphology to ensure accurate quantitative interpretation.

### 3.2 Electrochemical Analysis of WE43 in Various Environments Containing Protein

**Figure 2:**
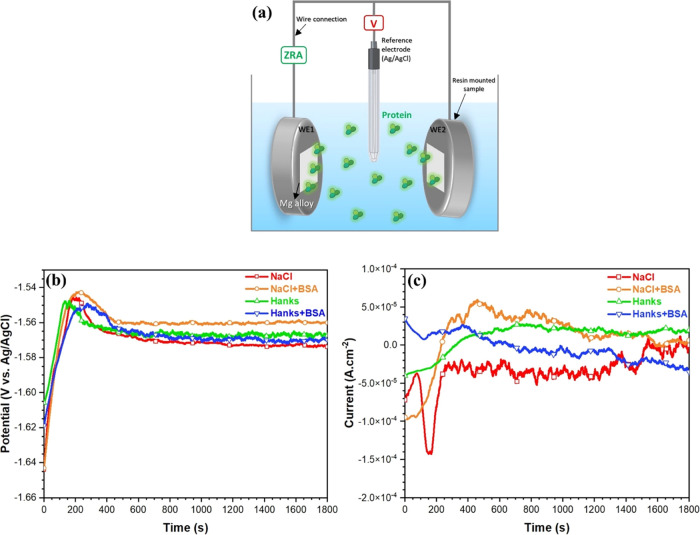
(a) Schematic representation of electrochemical noise setup. (b) EPN and (c) ECN signals of WE 43 alloy during immersion (30 min) in the different simulated physiological solutions with and without BSA protein. All measurements have been performed at 37 °C and pH 7.4.

Figure 2b,c shows the evolution of potential and current noise recorded for WE43 during 30 min of immersion in various solutions. Throughout the first 200–225 s, both time records for all solutions were unstable. The EPN shifted gradually (approximately 100 mV) to less negative values over the course of the first 400 s, reaching a nearly steady state. For the sample immersed in protein-free solutions (both NaCl and Hanks), the raw EPN signal rises gradually to approximately −1.55 V in the first 200 s, before decreasing sharply and stabilizing. The potential rise phase may be related to the formation of an oxide film and the subsequent potential drop of ∼30 mV can be attributed to the initiation of localized corrosion at the surface along with the formation of other Mg compounds.^46^

The effects of adding protein to NaCl and Hanks’ solutions are distinct. Adding BSA to the NaCl solution caused the film-forming potential to shift to more positive values (from −1.57 V for NaCl to −1.55 V for NaCl+BSA), whereas the effect of BSA addition on the Hanks’ EPN appears to be less pronounced, with only a slight shift in the negative direction observed (from −1.56 V for Hanks to −1.57 V for Hanks+BSA). During the same time, the density of the current fluctuated until a minimum peak was reached and then began to increase and stabilize. Based on the fluctuation amplitude of the ECN signal, different corrosion behaviors can be distinguished.^46^ The amplitude of the current fluctuations in the NaCl solution is greater than that of other solutions, and it increases with time (Figure 2c). It has been established that the amplitude of ECN is proportional to the corrosion intensity,^47^ which results in a higher double-layer capacitance of the corrosion product film (semiprotective and porous oxide film). As a result, it is possible to conclude that WE43’s passivity decreased and/or the corrosion process accelerated during the immersion period. BSA in NaCl has reduced current noise (lower amplitude) compared to BSA in Hanks. This is explained by the formation of a thick or multilayer of BSA protein (a strong metal–protein complex) with a lower electrical surface potential than the substrate, which tightly regulates the entire charge transfer for electrochemical interactions at the solid/protein/electrolyte interfaces.^16^ In contrast, the amplitude of the current noise increases slightly in Hanks containing BSA compared to Hanks without BSA due to the formation of an imperfect protective film and the heterogeneous distribution of phosphate and calcium phosphate products.^15,16,48^

**Figure 3:**
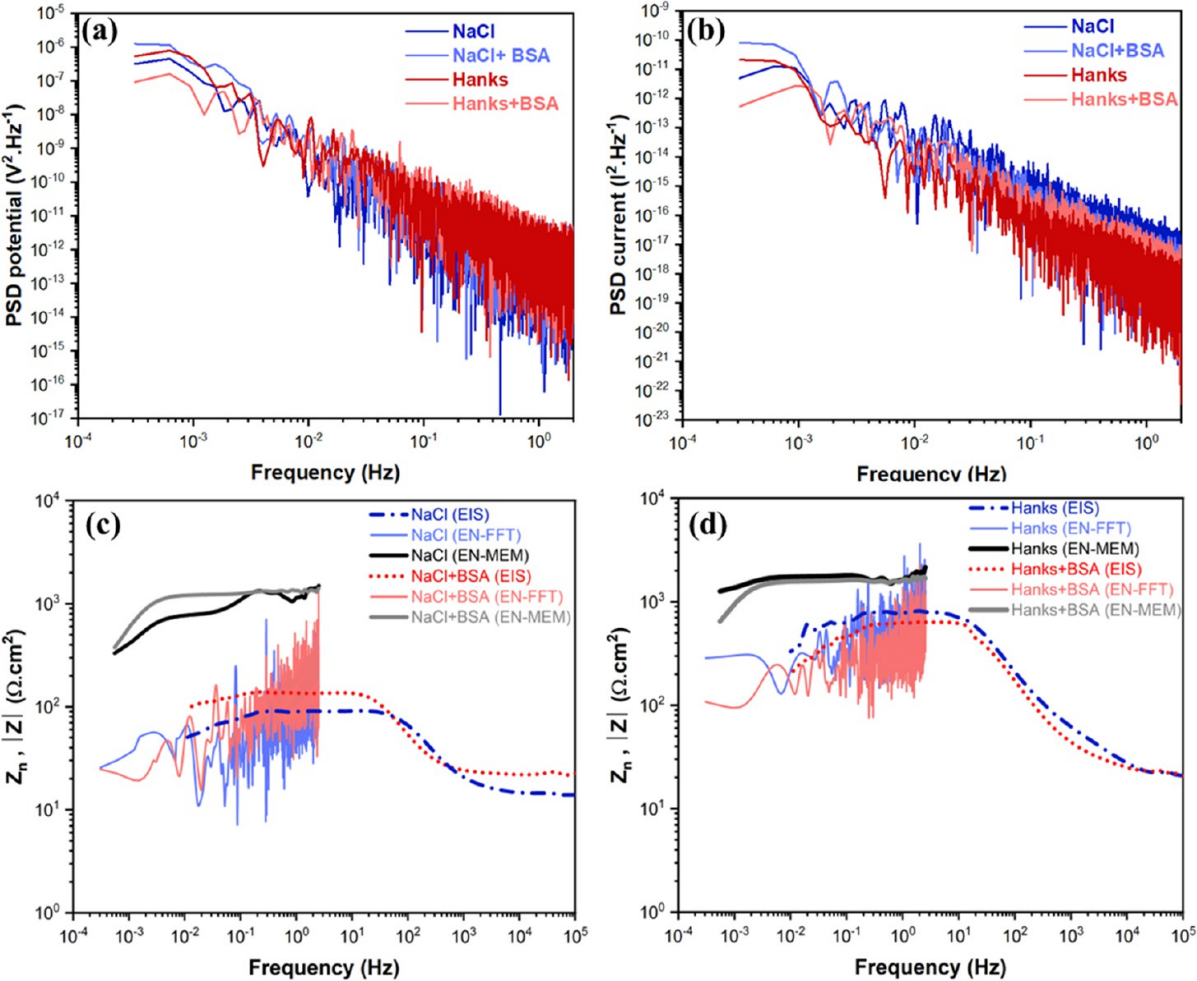
PSD analysis of (a) EPN and (b) ECN signals analyzed from Figure 2b,c. Impedance versus frequency graphs of WE 43 alloy in (c) NaCl and (d) Hanks’ solutions that were obtained by EN measurements in zero-resistance ammeter mode (*Z*_n_) and impedance moduli (|*Z*|) utilizing EIS at the OCP condition. All measurements were performed after immersion for 30 min in the different simulated physiological solutions with and without BSA protein at 37 °C and pH 7.4.

Figure 3 shows the extracted PSD analysis of the EPN and ECN signals in different physiological solutions. The corresponding slope of each PSD curve is known as the spectral power constant, which is related to the fractional Gaussian noise process and represents the properties of self-similarity and persistent stationary processes. The localized attack along the surface oxide layer may be responsible for the occurrence of repetitive potential and current transients in chloride-containing electrolytes.^49^ As shown in Figure 3a, the PSD of EPN signals reveals that the protective properties of the surface oxide layer deteriorate over time, as seen by the decreasing PSD across all solutions.^50^ Likewise, the slope of all PSD curves in both EPN and ECN signals (Figure 3a,b) has an approximately constant value throughout the entire frequency range. This particular feature (so-called white noise) in the frequency domain is normally assigned to a uniform corrosion process.^51^ The addition of BSA to NaCl causes the PSD slope of the EPN signal to be less steep compared to the solution without BSA, and adding protein to Hanks’ solution slightly increases the spectral power constant, indicative of a higher degradation rate, which supports the findings in Figure 2. Also, the addition of BSA to solutions containing NaCl and Hanks causes different behavior in the PSD magnitude of the ECN signals, resulting in a decrease in the NaCl solution and an increase in the Hanks’ media. It should be noted that although the four curves may appear to exhibit similar slopes, a rigorous analysis has been conducted to quantify the slope values for each condition. The results of this analysis demonstrate distinct slope values for different solution conditions. For instance, in Figure 3(a) (PSD potential), the measured slope values (from 10^–2^ Hz onward) are represented as −1.21 ± 0.07 for NaCl, −1.33 ± 0.04 for NaCl + BSA, −1.49 ± 0.04 for Hanks, and −1.46 ± 0.02 for Hanks+BSA. These values clearly illustrate the differences between the various solution conditions.

**Figure 4:**
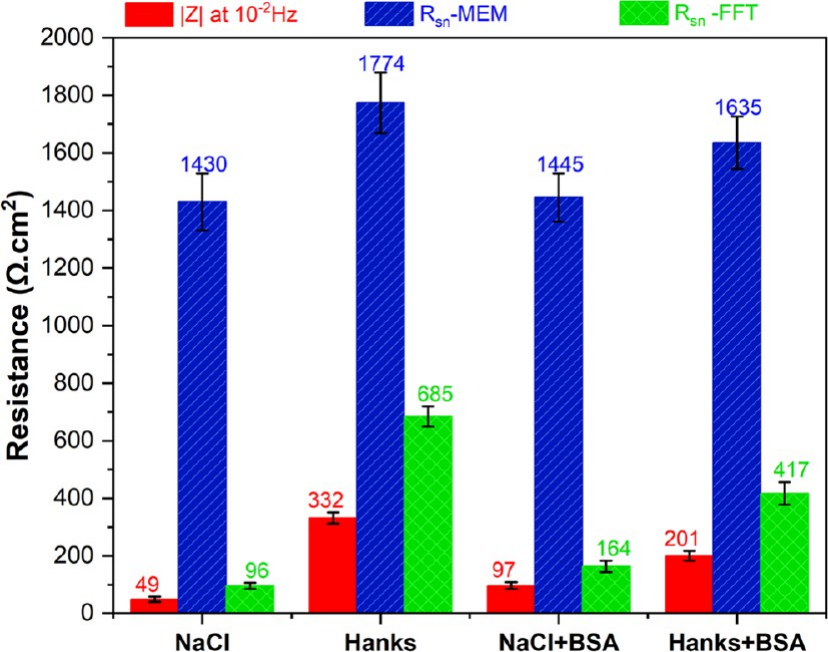
Extracted impedance values obtained by EN measurements and impedance moduli utilizing EIS shown in Figure 3.

Figure 3c,d demonstrates a comparison of the magnitude/Bode impedance diagram (amplitude current (AC) signal measurement) and noise resistance (direct current (DC) signal measurement) of WE43 when it was immersed in NaCl and Hanks’ solutions with and without the addition of BSA protein. The *Z*_n_ values were derived by using either FFT or MEM methods. For the NaCl solution, the polarization resistance, which is the sum of charge transfer resistance and protein complex film resistance (*R*_ct_ + *R*_protein film_), and the spectral noise resistance (*R*_sn_) increase with the addition of protein (Figure 4). These results indicate that BSA inhibits the corrosion of WE43 in the NaCl solution. From these data, a strong correlation can be established between polarization resistance (from EIS) and both noise resistance values (FFT and MEM methods) at low frequencies (Figure 4). However, the magnitude of noise resistance in both methods, especially at high frequencies, is greater than the corresponding polarization resistance value. Therefore, these results confirm that, despite the correlation between polarization resistance and noise resistance, they can not be considered the same parameter.^52^

### 3.3 Interaction of Inorganic Species and BSA Protein with the Mg Alloy Surface as Measured by XPS

**Figure 5:**
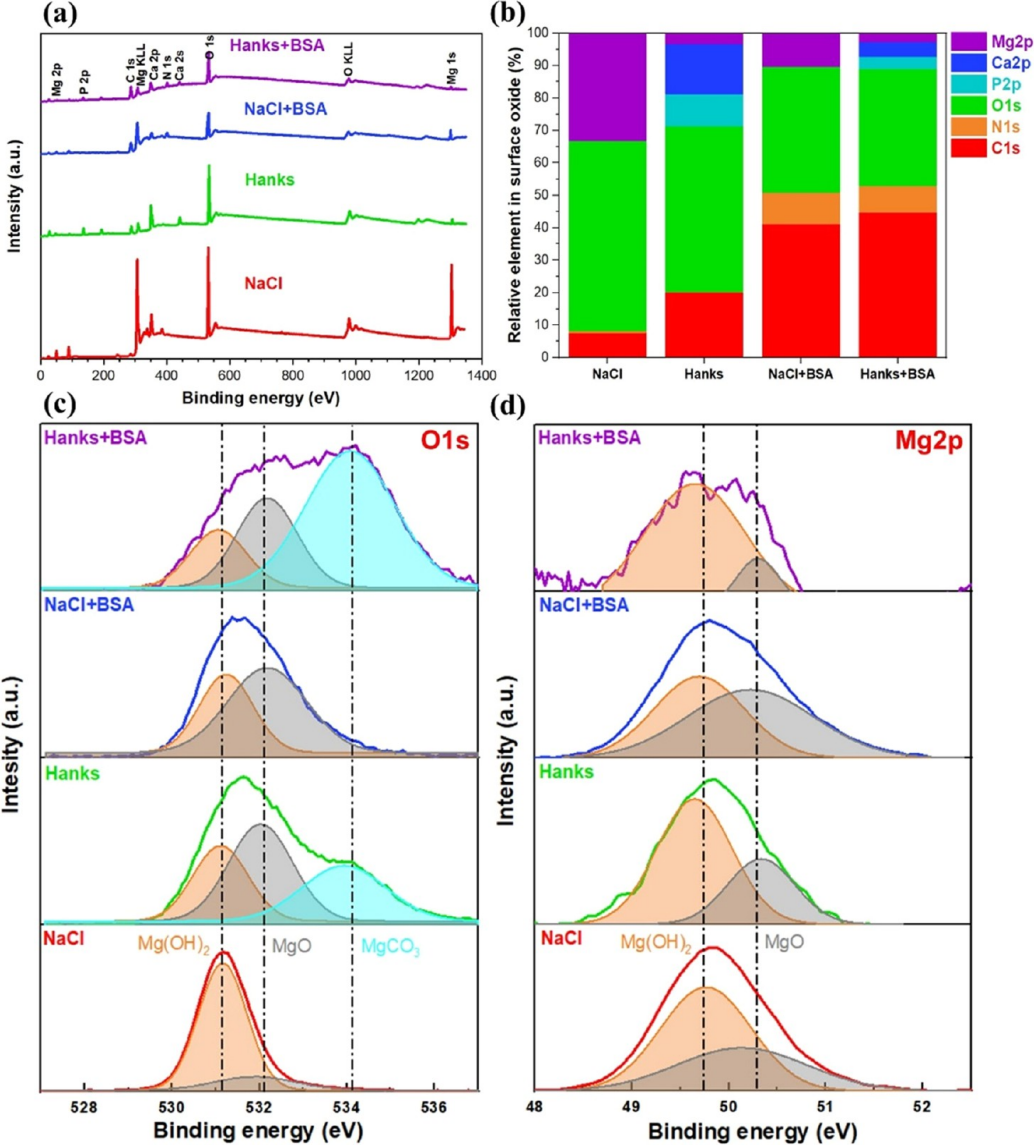
(a) XPS survey spectra, (b) relative percentage of elements in the surface oxide of the WE43 alloy calculated from the XPS survey spectra in (a), and (c, d) high-resolution XPS spectra of the O 1s and Mg 2p energy regions on the WE43 alloy after 10 min of immersion in 154 mM NaCl, and Hanks’ solutions with or without the BSA protein at pH 7.4 and 37 °C.

Figure 5a shows the measured survey spectra. Mg 2p peaks are seen between 48 and 52 eV in its high-resolution spectra (Figure 5d). These are associated with Mg(OH)_2_ (49.7 eV) and MgO (50.25 eV),.^16^ The oxide band is frequently present in all samples due to the formation of a thin MgO/Mg(OH)_2_ layer.^53^ The three peaks at 531.1, 532.1, and 534.2 eV in the O 1s spectrum (Figure 5c) correspond to chemisorbed hydroxide (Mg(OH)_2_), oxide (MgO), and magnesium carbonate (MgCO_3_), respectively.^41,54^ The majority of the C peak on all specimens (more pronounced on surfaces lacking protein interaction) was due to air contamination.

According to ref (6), the albumin protein’s molecular structure consists of carboxyl, peptide, and amino groups. Accordingly, it is feasible to deconvolute three separate bands in C 1s, containing 285, 286, and 288 eV. Furthermore, C 1s signals between 289 and 290 eV reveal the presence of CO_3_^2–^ on the surface of the samples as a result of the biodegradation processes that produce MgCO_3_ and CaCO_3_.^53^ As a result, the increased intensity of C 1s peaks in the corrosion product layer of the WE43 exposed to the albumin protein, as well as the presence of N 1s peaks, are associated with protein adsorption/complex formation.^16^ To better visualize the proportion of protein adsorption on the WE43’s surface in the two different environments, a comparison of the relative atomic ratio between N (N 1s) and the oxidized carbon C 1s ([N/(C2+C3)]) peaks was evaluated. This atomic ratio represents the amount of adsorbed BSA protein on the Mg oxide layer which is ∼0.24 for NaCl and ∼0.18 in the Hanks’ environment.

Magnesium phosphate and calcium hydroxyapatite are associated with the P 2p spectrum at *ca*. 133 eV.^55^ Based on the XPS spectra, Figure 5b presents the elemental distribution in the Mg surface oxide. Switching from NaCl to Hanks’ solutions, the Mg and O signal intensities in the oxide layer or the corrosion products of the sample were moderately reduced. In addition, the layer produced by the Hanks’ solution is rich in calcium and phosphate components. Moreover, the amount of Mg in the corrosion products was reduced as a result of BSA protein being added to all solutions. In agreement with the electrochemical observations, it has been reported that promoting the formation of hydroxide, phosphate, and calcium phosphate compounds can significantly reduce metal ion release and, thus, increase the corrosion resistance of the alloy.^48^ Hydroxyapatite is formed in Hanks’ solution due to the preferential interaction of phosphate species with Ca^2+^ at near-neutral pH.^10^ Carbonate products in the electrolyte that may cover the surface of the Mg alloy due to the unusual interaction of Ca^2+^ and HCO_3_^–^ species in the Hanks’ environment may impede biodegradation processes.^48^

Noise measurements and XPS data demonstrate that the increased corrosion resistance is accompanied by increased protein adsorption on Mg in NaCl. This phenomenon can be explained by the formation of a thick or multilayer of the BSA protein, also known as a strong metal–protein complex.^16^ This layer has a lower electronic conductivity (less surface potential and/or surface charge) than the substrate, and it strongly controls the whole charge transfer for the electrochemical interaction that takes place at the interfaces.^56^ The presence of BSA in Hanks caused a modest decrease in the corrosion resistance of WE43 by diminishing the Ca/P and P intensity signals and, in particular, fostering metal-protein complex formation. Due to the defective and thin protective barrier, and nonhomogeneous distribution of phosphate products, the self-protecting effect of these species against corrosion was attenuated in BSA protein media.^16^

### 3.4 Morphological and Surface Potential Evolution of WE43 in Different Environments Containing Protein

**Figure 6:**
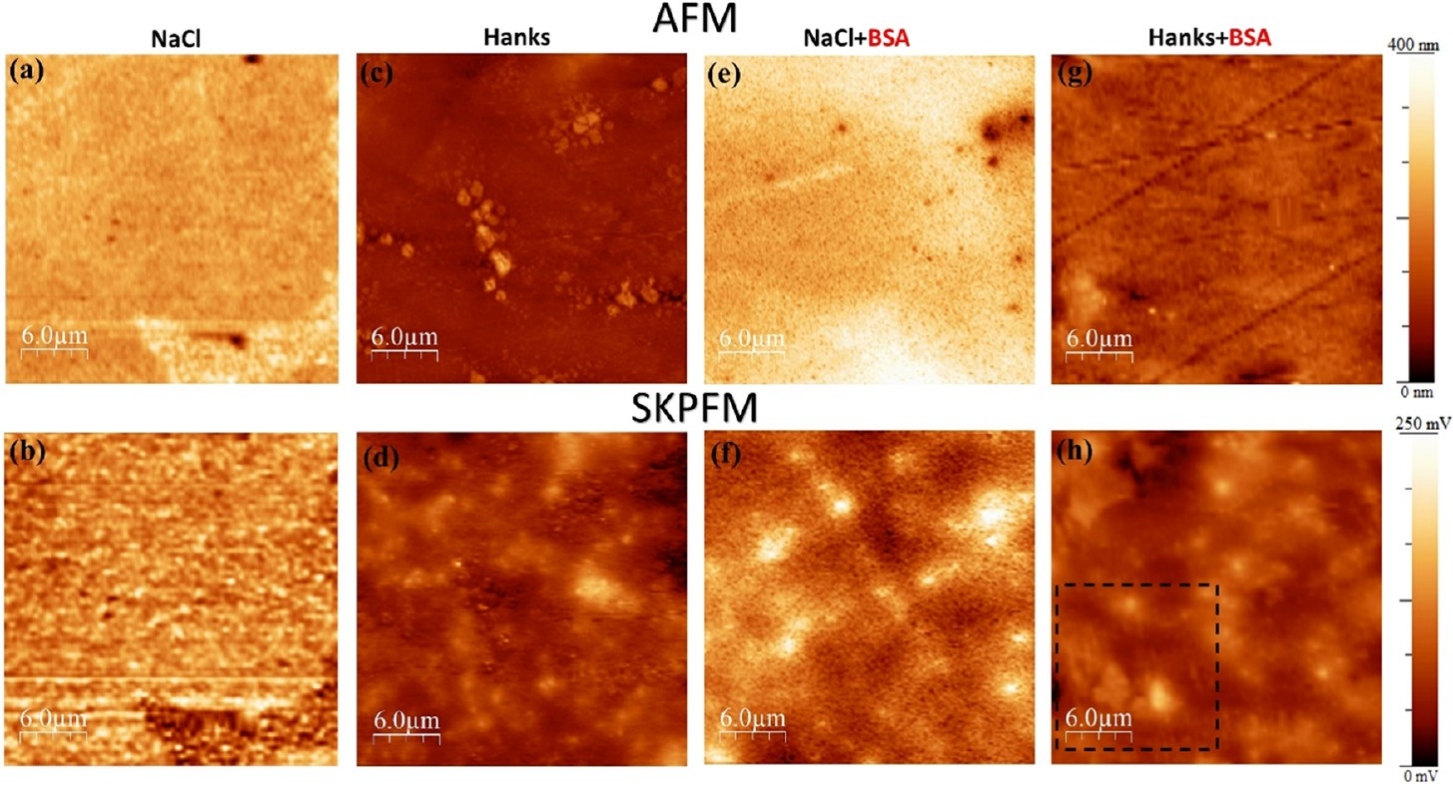
AFM (first row) and SKPFM (second row) images of WE 43 alloy after 10 min immersion in (a, b and e, f) 154 mM NaCl and (c, d and g, h) Hanks’ physiological solutions with or without the BSA protein at 37 °C and pH 7.4.

**Figure 7:**
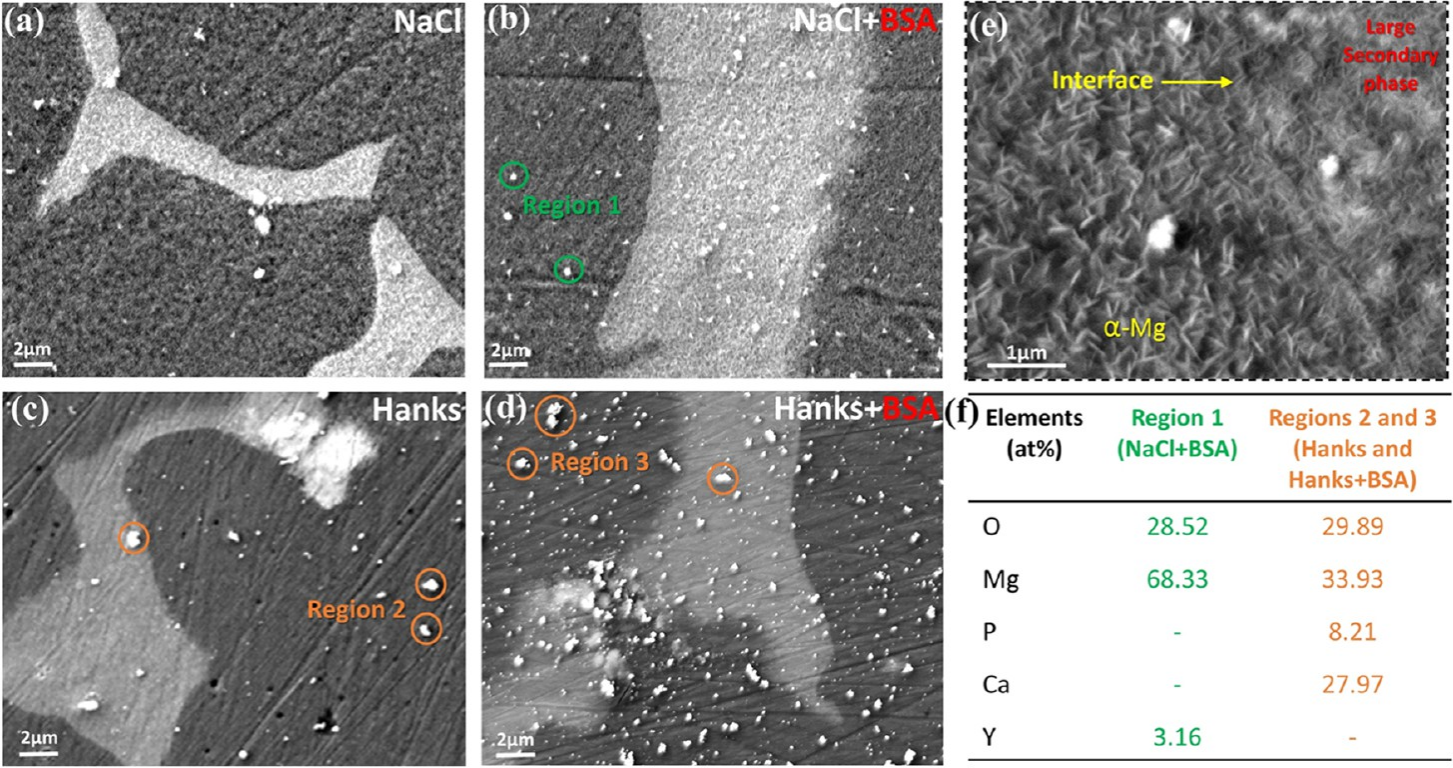
SEM images of WE 43 alloy after a 10 min immersion in (a) 154 mM NaCl and (b) 154 mM NaCl + 4g/L BSA, (c) Hanks’ and (d) Hanks’+4g/L BSA physiological solutions at 37 °C and pH 7.4. (e) Magnified image of secondary phase/α-Mg (matrix) interface in NaCl solution. (f) Average values of multi-EDXS analyses of bright white spots in (b), (c), and (d).

The rate of metal ion release or degradation, the type of corrosion products, and especially the formation of a protective layer on the surface of Mg and its alloys are all highly sensitive to the chemistry of the solution, pH, as well as the type and concentration of ions and inorganic/organic species.^10^ Furthermore, the distribution of charged and polar residues in the protein molecular structure and its isoelectric point are drastically impacted by these parameters, which directly regulate the nature of protein physicochemical interactions and their adsorption mechanisms.^7^ The combined AFM and SKPFM surface analyses have been used to visualize WE43’s topography and electrical surface potential distribution during the initial stages of immersion (first 10 min). Figure 6 shows the topography and electrical surface potential maps of WE43 in NaCl and Hanks’ solutions with and without the addition of BSA. In the NaCl solution, corrosion is uniform on both matrix and secondary phases as per the AFM topography and its corresponding electrical surface potential map from SKPFM (Figure 6a,b). This indicates that the Mg matrix is slightly more corroded than the secondary phases, which is further confirmed by the SEM images in Figure 7a,e. As seen in Figure 6b, the electrical surface potential of the Mg oxide film or corrosion products in secondary phases is less than that for the Mg matrix, and its magnitude is the opposite of that of the fresh surface. So, the formation of rare-earth corrosion products in the secondary phases of WE43 that have distinct electronic properties (e.g., WFE, n-, or p-type semiconductor characters) seems to significantly modify the surface potential magnitude.

Nonetheless, in the Hanks’ solution, the AFM image and SKPFM map (Figure 6c,d) display heterogeneous topographies and electrical surface potential distributions without any evident indications of secondary phases. However, these phases can still be detected by SEM analysis (Figure 7c). When protein is present in an electrolyte, the AFM topography maps are notably distinct from those viewed when the protein is absent. Particularly, in the case of the NaCl solution, the secondary phases are not readily apparent in the topography image and the related SKPFM map represents only a heterogeneous electrical surface potential distribution due to the formation of diverse metal-protein complexes.^16^ The surface potential map for the sample immersed in Hanks’ solution containing BSA protein showed a heterogeneous pattern of novel surface features with a lower electrical surface potential and/or surface charge than the Mg oxide layer (Figure 6h). According to the previous investigation, these novel surface characteristics consist of nanolayers of the adsorbed protein with aggregated and/or fibrillar structures.^16^ The total electrical surface charge distribution in the molecular structure of soft biological materials such as proteins typically governs the electrical surface potential of these substances. Depending on the ionization state of protein amino acid groups, a protein molecule can show neutral, negative, or positive charges.^56^

**Figure 8:**
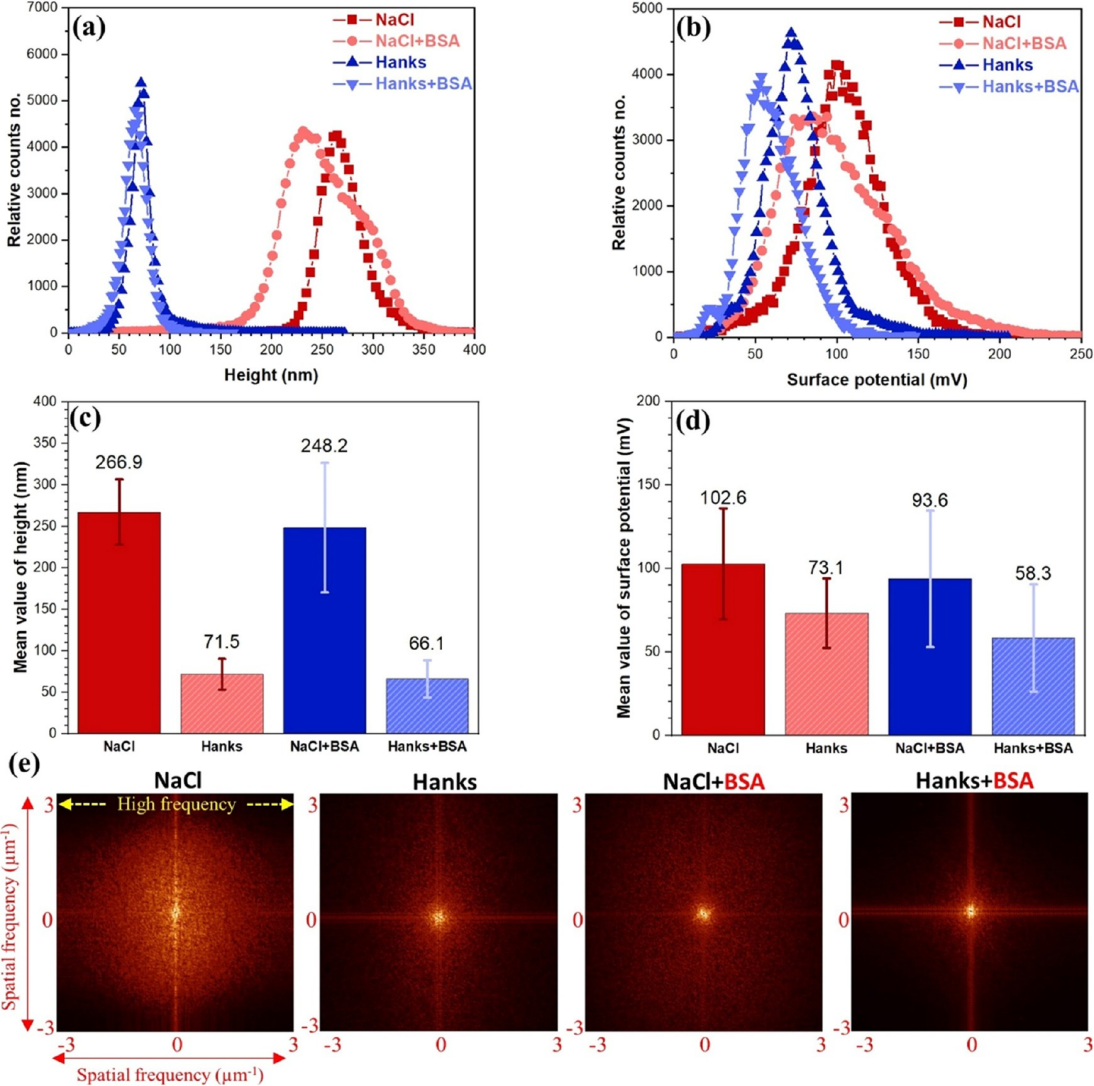
(a) Topography and (b) surface potential histograms of WE 43 alloy obtained from AFM and SKPFM images in Figure 6. (c, d) Extracted Gaussian distribution from the topography and surface potential histograms in (a) and (b). (e) 2D PSD analysis of surface potential maps in Figure 6.

The SKPFM maps of the samples after adding BSA to the solution are noticeably different from those seen in the unmodified solution (Figure 6). The histographic distributions of surface roughness^57^ and electrical surface potential were taken from Figure 6 and displayed in Figure 8 to better comprehend the effect of both inorganic species in Hanks’ media and protein molecules. Incorporating BSA into the NaCl solution led the SKPFM histogram to display a heterogeneous (due to a continuous network of dense protein or cluster domains) and a lower surface potential distribution than a blank NaCl solution. According to the histographic analysis of the surface potential maps, the total surface potential deviation (standard deviation in a Gaussian fit^58^) on the surface of the alloy in the Hanks + BSA condition is marginally larger than that of the sample in the Hanks’ solution without protein (Figure 8d). This rise in the surface potential deviation indicates that protein adsorption on the surface of the Mg alloy increases the heterogeneity of surface potential distribution.

In addition, the 2D PSD results presented in Figure 8e reveal a reduced surface potential distribution on the WE43 alloy surface under the Hanks + BSA condition at nearly all spatial frequencies. Based on the histogram and PSD analyses, the surface potential difference for the sample immersed in the Hanks+BSA solution is lower than that of the sample exposed in the plain Hanks’ solution for all spatial frequency ranges. This proves that proteins have been adsorbed to the surface of the sample, even though protein clusters have not been seen.^7^ Compared to the Hanks+BSA solution, which exhibited a semihomogeneous distribution of surface potential due to the dominating BSA protein area covering the matrix, the distribution of surface potential of all constituents was found to be less uniform in the NaCl-containing protein condition (Figure 8b,e). Moreover, histographic analysis of the topographical maps in Figure 8a further shows that the surface roughness of the sample immersed in Hanks’ solution shifts to a lower value, roughly ∼196 nm, compared to that of the samples immersed in the NaCl solution (Figure 8c). The semiprotective corrosion products growth during immersion, and the significant role of the covered layer of BSA proteins, carbonate, and phosphate species, are likely responsible for the lower surface roughness distribution on the Mg alloy surface in the Hanks’ and Hanks + BSA exposure conditions compared to that in NaCl and NaCl+BSA solutions (Figure 8a,c).

**Figure 9:**
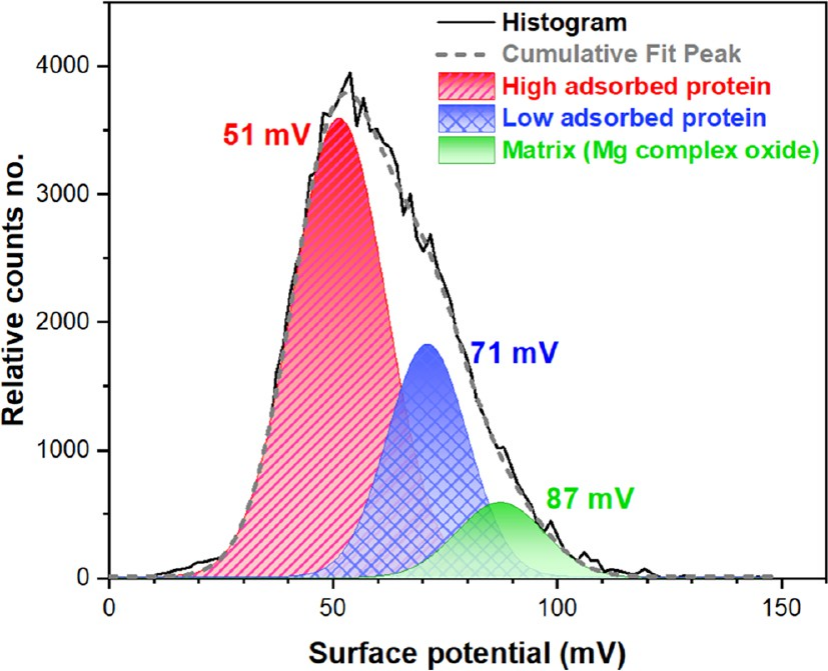
Example of a simulated multinomial (trimodal) Gaussian distribution of the histogram plot corresponding to Figure 8b (Hanks+BSA media).

Figure 9 presents a deconvolution of the surface potential histogram related to the Mg alloy in Hanks+BSA media to identify the distribution of electrical surface potential of individual surface components in multiple modes. The results show that the Mg matrix has the highest surface potential value (approximately 87 mV), whereas the areas with distinct protein adsorption (i.e., high-adsorbed and low-adsorbed) have lower mean values of surface potential compared to the matrix. The versatility of the Kelvin probe method lies in its ability to measure the work function of various materials under diverse experimental conditions. This sets it apart from other surface techniques that have limited applicability.^5^ The total WFE, the multipoles of the surface components, and the static charges are all strongly related to the surface potential or electrostatic interactions in any system of semiconductor or dielectric materials.^56^ In WE43, for instance, the electrical surface potential signal on the oxide layer is determined by the combined WFE of the oxide components, which is in turn determined by their weighted concentrations.^16^ Even so, the electrostatic interactions and charge transfer process, in particular at the protein nanobiofilm/oxide layer interface, are heavily influenced by certain physical and chemical properties of the substrate and oxide layer, such as the surface roughness, charge carriers, charge distribution, surface energy, crystallinity and texture, and conduction and valence bands.^7,59^

**Figure 10:**
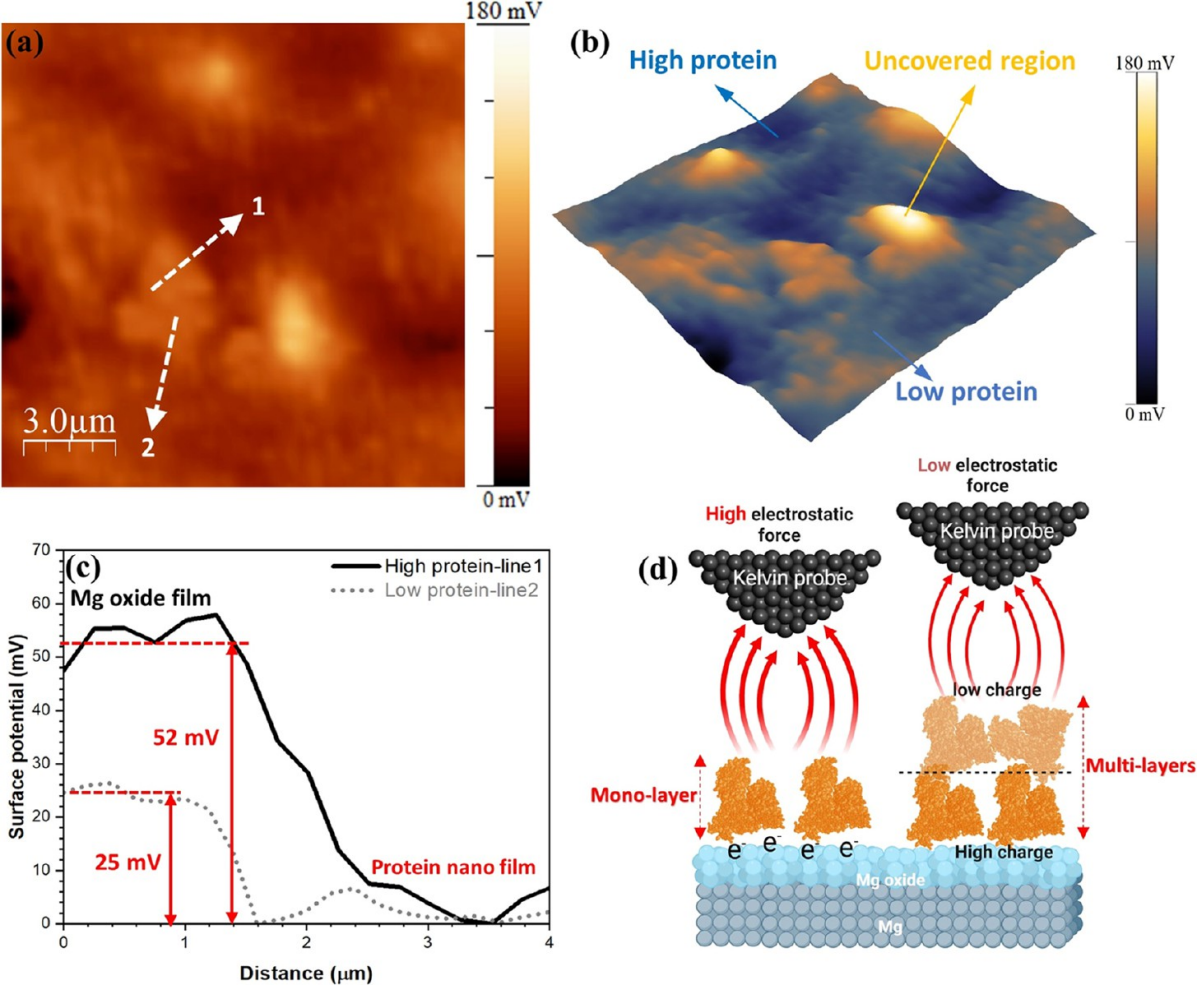
(a) Magnified-surface potential map of WE 43 alloy in Hanks’ solution containing BSA protein obtained from Figure 6(h). (b) 3D presentation of the surface potential map with various surface features related to (a). (c) surface potential line profiles related to (a). (d) Schematic representation of electrostatic interaction between a conductive AFM tip-apex and mono- or multilayers of adsorbed protein on Mg oxide layer at the atomic scale.

It is well known that the observed surface potential is considerably influenced by the adsorption of monolayers or multilayers of organic molecules on a metallic substrate in physiological fluids.^5^ This is shown schematically in Figure 10d for BSA molecules on the Mg oxide surface. This figure represents that the electrostatic interaction between the conductive tip of the SKPFM and the adsorbed BSA molecule on the oxide layer is altered as a result of the BSA molecule’s attachment to the oxide surface. BSA’s interaction with the oxide layer’s interface causes band bending on the protein molecule side of the energy band diagram, which in turn changes the effective molecular dipole and interface dipoles.^56^ This shift in the energy band diagram is due to the reorganization and redistribution of charge carriers in the BSA-adsorbed portion of the oxide layer.^60^ As a result, the magnitude of the electrical surface potential on the BSA molecule-complex oxide is susceptible to all of the aforementioned factors.^61^ Also, the contribution of the bulk material on the total surface potential is significantly mitigated by the formation of a thick organic film (>100 nm) on the surface oxide layer due to the limited range of interactions between the tip and the studied surface (metal/oxide film in this work).^5^

Nanoscale SKPFM (2D and 3D) surface maps were obtained, and they are shown in Figure 10a,b. These images demonstrate the desaturated structure of the BSA molecule absorbed on the oxide layer with a heterogeneous surface potential or charge distribution. The surface potential of biological molecules is highly dependent on charge distribution and polar residue structure, in particular pH and isoelectric point (pI).^62^ The pH of a solution at which the net charge of a protein equals zero is known as the pI. Since the protein surface is predominately negatively charged at solution pHs above the pI (dissolution of Mg in physiological media increases the pH), like-charged protein molecules will display repulsive forces.^63^ Theoretical modeling and experimental investigations estimate the pI value of the BSA protein to be between 4.7 and 5.4.^56^

Figure 10c shows line profiles of the electrical surface potential, which indicate that the surface potential and/or surface charge distribution on the BSA molecule structure are approximately 52 mV lower than on the complex oxide layer on WE43. Because of the presence of additional potential steps and band bending at the energy level, the electrical surface potential was reduced upon chemisorption of the BSA molecules on the Mg oxide layer, as was previously indicated. Consequently, this nanoscale surface potential difference demonstrates the BSA protein’s inhibitory effect on the surface potential/charge distribution, which, in turn, affects the electrochemical interaction at the BSA molecule/oxide layer interface, as discussed in the preceding sections. Figure 10d shows that as the number of protein layers adsorbed onto the oxide film surface increases from a monolayer to multiple layers, the misalignment in energy levels (more band bending) increases, resulting in a reduced electrostatic force and a lower potential difference between the tip and the protein-oxide surface. The monolayer of BSA protein has a more substantial charge distribution at the protein/oxide film interface than subsequent protein layers (low-protein and high-protein line profiles in Figure 10c).^62^ It is crucial to note that the structure of BSA molecules has a lower electrical charge transport function compared to other proteins, such as Azurin and bacteriorhodopsin, and this has a significant impact on the electron transfer process in the protein–protein interactions.^56^

**Figure 11:**
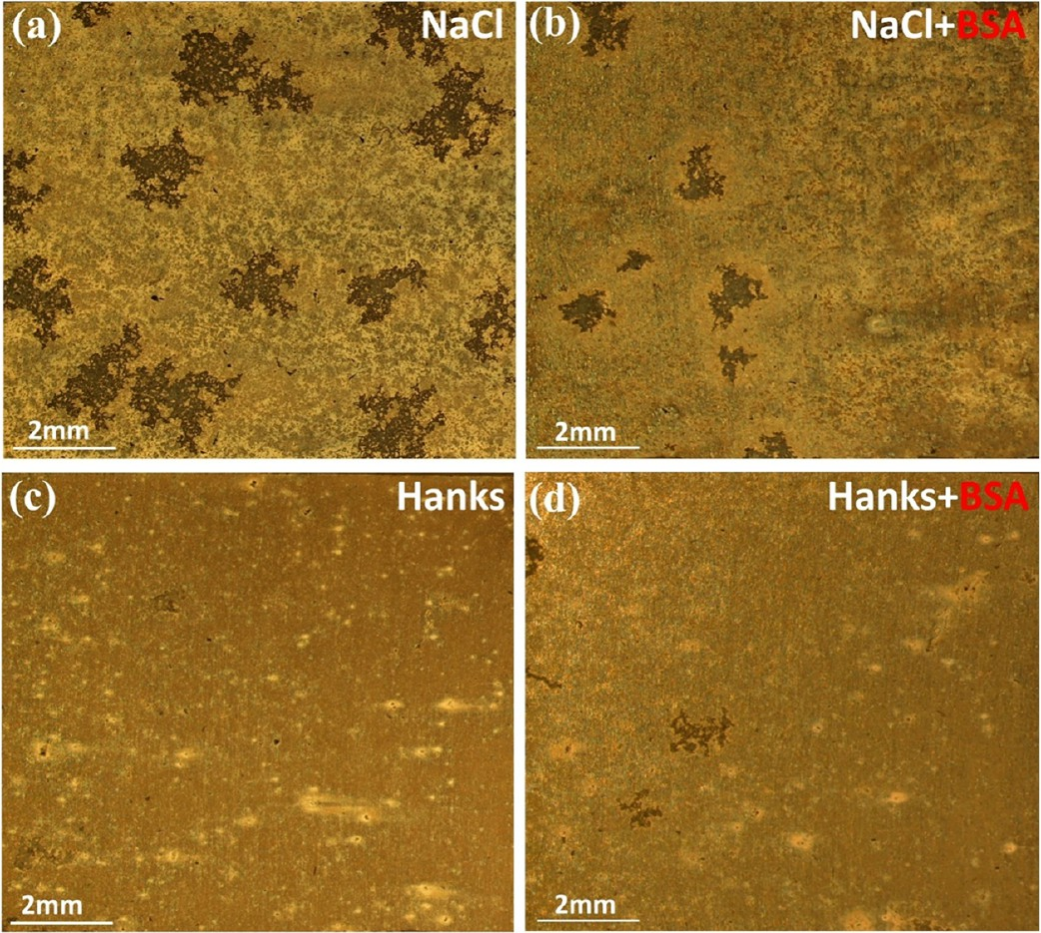
Optical microscopy images of WE 43 alloy after 30 min immersion in (a) 154 mM NaCl, (b) 154 mM NaCl+BSA, (c) Hanks, and (d) Hanks+BSA at 37 °C, pH 7.4, and aerated conditions.

Figure 11 presents optical microscope images of the surface of WE43 after 30 min of immersion in various solutions. Consistent with earlier findings in this study, the NaCl solution (Figure 11a) resulted in a significantly higher degree of localized corrosion on the surface than that of the Hanks’ solution. Figure 11b demonstrates that the inclusion of BSA inhibits the degradation of WE43 in NaCl. In the presence of protein, fewer corrosion initiation sites were found. Also, there are fewer “craters” on the surface. These craters are formed due to the simultaneous reduction of water and the formation of H_2_ bubbles upon immersion. This leads to the formation of localized alkaline regions due to the release of hydroxide groups and shows the presence of cathodic sites at the intermetallic phases just beneath the corrosion layer.^15,64^ It is consistent with the reported shape of the corrosion layer on WE43 that forms during immersion in various body fluids^9,15,65,66^ to link the formation of localized areas to the underside of cathodic intermetallic sites. In contrast, the addition of BSA to Hanks’ (Figure 11d) somewhat accelerates the surface deterioration process due to competition between inorganic species in Hanks’ media and protein molecules, which reduces the inhibitory impact of BSA. Furthermore, in the Hanks’ solution containing BSA protein, the self-protective activity of phosphate and calcium phosphate species against corrosion and biodegradation processes was reduced.^16^

**Figure 12:**
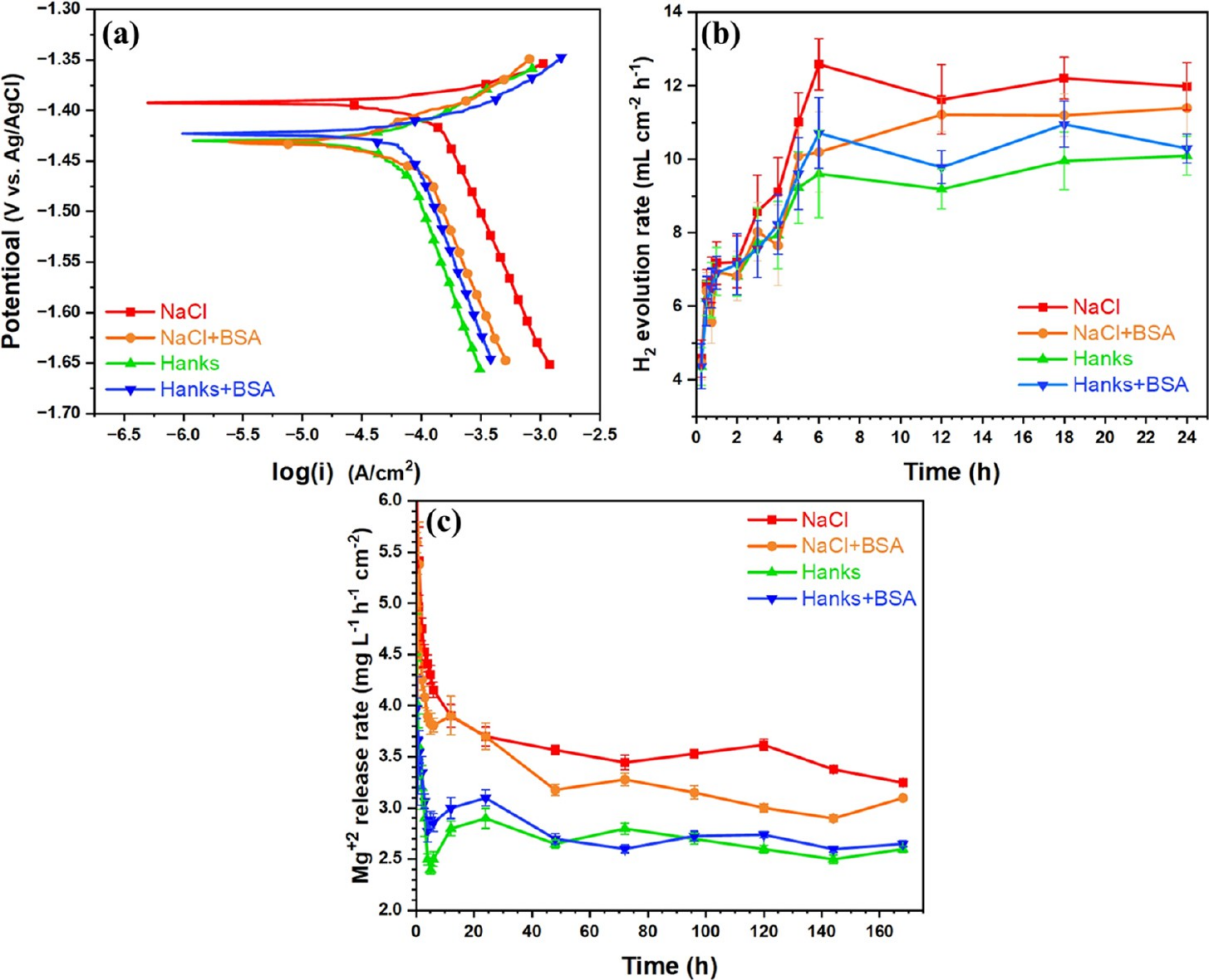
(a) Potentiodynamic polarization, (b) Hydrogen evolution rate, and (c) release rate of Mg^2+^ values for WE43 immersed in NaCl, NaCl + BSA (4 g L^–1^), Hanks, and Hanks + BSA (4 g L^–1^). Data presented with standard deviation.

In conjunction with other findings, measurements of the linear polarization resistance and the rate of hydrogen evolution reveal the corrosion reaction kinetics, as shown in Figure 12 and Table S1. The polarization resistance (Rp) serves as an inverse indicator of the degradation rate and can be determined using the Stern-Geary method (refer to the Supporting Information).^15,67^ The anodic and cathodic branches of the curves in Figure 12a define the kinetics of the anodic dissolution and cathodic hydrogen evolution reactions, respectively. The curves illustrate that the addition of BSA reduces the kinetics of the cathodic hydrogen evolution reaction in a NaCl solution. Compared to Hanks’ solution, when protein is added, the anodic branch shifts toward a slightly higher current density, indicating enhanced anodic activity attributed to reduced barrier resistance against the infiltration of aggressive ions.^31,32^ This is also evident in the corrosion current density (*j*_corr_) values: *j*_corr_ is at its lowest in Hanks and highest in NaCl (Table S1).

The hydrogen evolution rate (HER) serves as a proxy for the corrosion rate as both occur at the same rate.^15,32^ The HER (Figure 12b) was consistently fastest in NaCl and lowest in Hanks during the initial 6 h of immersion. After 6 h, the HER rate decreases, suggesting the absence of a fresh surface, likely due to the formation of an adsorption layer alongside a complex corrosion product. Previous studies have demonstrated that proteins and other organic components can synergistically form a dense adsorption layer, effectively reducing the corrosion rate of Mg in saline solutions.^16^ Between 1 and 6 h of immersion, the HER increases in all four media, gradually decreasing until 24 h.

To further investigate the role of proteins in the corrosion behavior of the WE43 alloy, the rate of Mg release was also evaluated. During the initial stage of immersion (up to 30 min), there was a rapid release of Mg^2+^ ions into each of the four solutions (Figure 12c); however, consistent with the electrochemical findings, the release rates were slower in Hanks’ solutions. After 30 min, the release rate notably decreases, presumably due to the development of an inorganic-based corrosion product film on the alloy surface.^68^ Between 6 and 48 h, the Mg^2+^ release rate in Hanks’ solution without BSA was significantly lower than in the NaCl solutions. After 168 h, the release rate became relatively low and similar across all tested environments, indicating complete coverage of the alloy surface with corrosion products.

**Figure 13:**
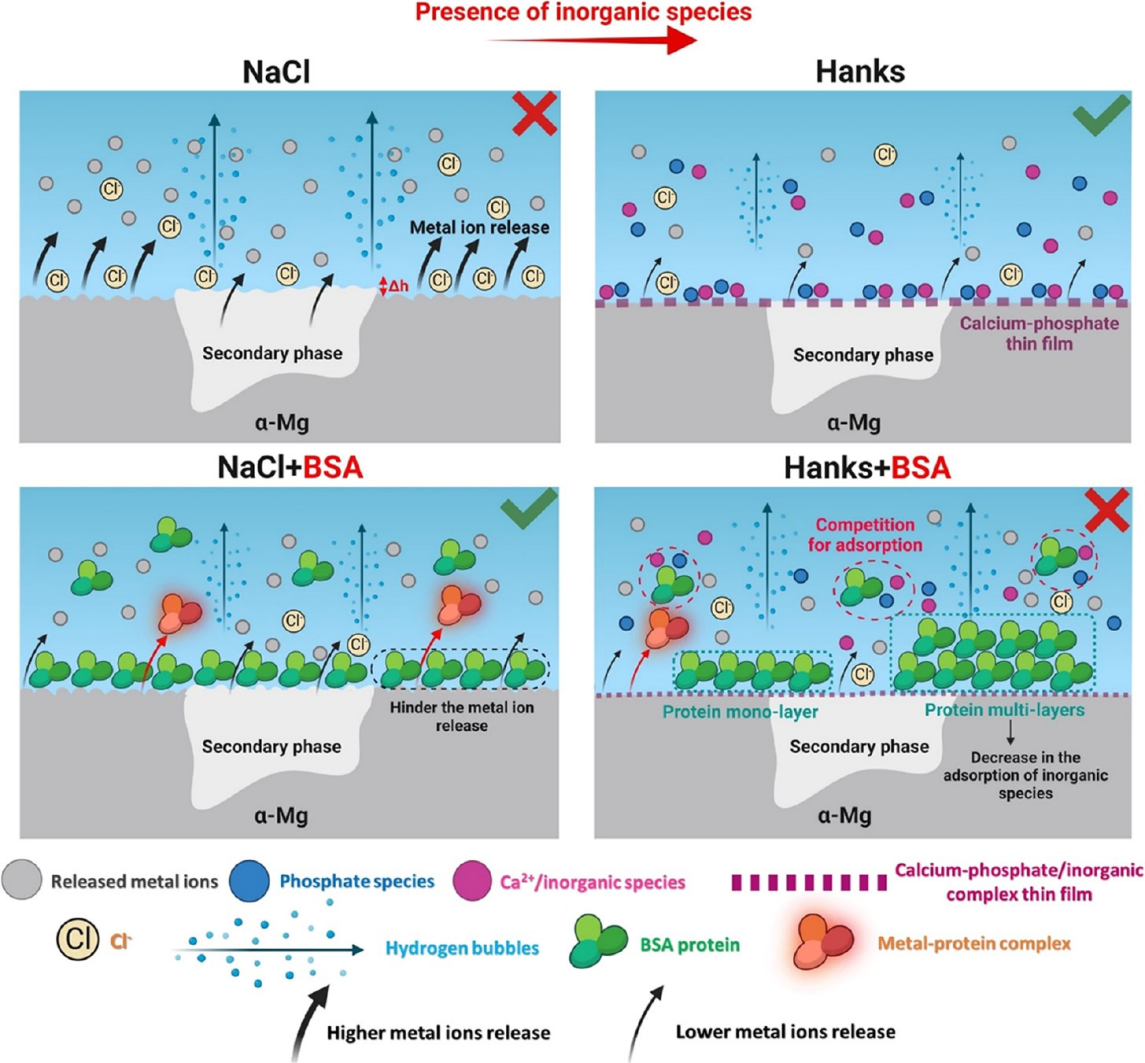
Summarized schematic illustration of the role of BSA proteins and inorganic species on the early-stage biodegradation process of the WE43 alloy in different simulated physiological solutions. The red and green signs, respectively, present the high (detrimental impact) and low (beneficial impact) corrosion and biodegradation processes of Mg alloy in different environments.

The impact of inorganic and organic substances on the corrosion of the WE43 alloy in NaCl and Hanks’ solutions is depicted in Figure 13. Inorganic species, notably calcium phosphate and other complex thin films, effectively reduce uniform corrosion and hinder localized corrosion.^10,69^ However, rapid anodic dissolution and hydrogen evolution were observed in NaCl media without complex inorganic species. By introducing biological organic species, such as protein molecules, a dual-mode biodegradation process can be observed.^70^ In NaCl, a higher protein surface coverage reduces the level of Mg degradation and hydrogen evolution. Conversely, in Hanks, interactions between protein molecules and inorganic species lead to a lower protein coverage. Furthermore, in NaCl, the rough surface makes the visualization of the protein film challenging, while in Hanks, interactions of protein molecules with inorganic species affect zeta potential and the formation of regions with low and high protein coverage, which can be detected using SKPFM.^6^

## 4 Conclusions

In conclusion, our study used a combination of DC and AC multielectrochemical analyses, X-ray photoelectron spectroscopy, and atomic force microscopy with scanning Kelvin probe force microscopy (AFM/SKPFM) to investigate the corrosion and biodegradation behaviors of WE43 in various solution conditions and in the presence of protein molecules. Our key findings are as follows:

Alloy Environment: WE43 alloy’s corrosion rate is significantly reduced in complex inorganic solutions compared to NaCl, highlighting the importance of understanding the ionic composition of the environment for alloy design. This insight can lead to more durable devices and warrants further exploration in the field of Mg-based alloys.

Protein Effects: The influence of the protein on corrosion varies with solution chemistry. Bovine serum albumin appears to act as a corrosion inhibitor in NaCl but accelerates biodegradation in Hanks’ solution. These findings contribute to foundational knowledge and can enhance predictive models and corrosion control strategies.

Non-Uniform Protein Adsorption: AFM/SKPFM revealed a non-uniform protein adsorption process on the alloy surface, emphasizing the complexity of corrosion. This nonhomogeneous adsorption highlights the role of protein distribution in the corrosion mechanism.

In summary, our research has far-reaching implications, offering insights into alloy design, corrosion control, and protein-induced corrosion. These findings have broad applications across diverse fields, from healthcare to aerospace, and beyond.

## Supporting Information Available

Experimental information for the polarization resistance fitting process (PDF)
