# Supplementary material for: Albumin Protein Impact on Early-Stage In Vitro Biodegradation of Magnesium Alloy (WE43)
Source: ACS Appl Mater Interfaces. 2023 Dec 18;16(1):1659–74. doi: 10.1021/acsami.3c12381 (PMC10788864; doi:10.1021/acsami.3c12381)
Supplement: Supplementary file 1 — am3c12381_si_001.pdf [file am3c12381_si_001.pdf]

Supporting information

Albumin Protein Impact on Early-Stage *in vitro* Biodegradation of Magnesium Alloy (WE43)

Amin Imani[a,b*], Ehsan Rahimi[c**], Maria Lekka[d], Francesco Andreatta[e], Michele Magnan[e], Yaiza Gonzalez-Garcia[c], Arjan Mol[c], R.K. Singh Raman[b,f], Lorenzo Fedrizzi[e], Edouard Asselin[a]

[a]Department of Materials Engineering, The University of British Columbia, Vancouver, BC, V6T 1Z4, Canada

[b]Department of Mechanical and Aerospace Engineering, Monash University, Clayton, VIC 3800, Australia

[c]Delft University of Technology, Department of Materials Science and Engineering, Mekelweg 2, 2628 CD Delft, The Netherlands

[d]CIDETEC, Basque Research and Technology Alliance (BRTA), 20014 Donostia, San Sebastián, Spain

[e]Polytechnic Department of Engineering and Architecture, University of Udine, 33100 Udine, Italy

[f]Department of Chemical and Biological Engineering, Monash University, Clayton, VIC 3800, Australia

Corresponding authors:

[*]A.Imani: amin.imani@ubc.ca , amin.imani@monash.edu

[**]E.Rahimi: e.rahimi-2@tudelft.nl

Determination of polarization resistance by using the Stern-Geary equation:

In Mg degradation, due to the copious hydrogen evolution during polarization, both the cathodic and anodic sides create ohmic gradient drops, which are difficult to compensate for [1]. Therefore, the determination of the Mg corrosion rate by PDP should be done with caution and should be augmented by other methods (e.g., Hydrogen evolution rate measurements).

The electrochemical parameters from potentiodynamic polarization (corrosion potential (E_{corr}), corrosion current density (J_{corr}), cathodic/anodic Tafel slopes (β_c and β_a)) were taken directly from the PDP curves. The polarization resistance (R_p) was derived based on the region of the curve where current density is a linear function of the applied potential, near E_{corr}. R_p is inversely proportional to the Mg degradation rate [2–4]:

$$R_p = \frac{\beta_a \times \beta_c}{2.303 j_{corr}(\beta_a + \beta_c)}$$

Tafel slopes are calculated by assuming activation-controlled kinetics of cathodic and anodic half-cell reactions for Mg at potentials far enough from E_{corr}.

Table S1. Fitted potentiodynamic polarization variables extracted by the Tafel extrapolation for Mg alloy WE43 after immersion at 37 ± 0.5°C in NaCl, NaCl + BSA, Hanks, Hanks + BSA; E_{corr}: corrosion potential; j_{corr}: corrosion current density; R_p: polarization resistance.

**Solution**	E_{corr} [$V_{Ag/AgCl}$]	j_{corr} [$\mu A/cm^2$]	*CR* [mm/year]*	R_p [Ω.cm]
**NaCl**	-1.39	126.8	3.5	114
**NaCl + BSA**	-1.43	97.9	2.7	200
**Hanks**	-1.42	74.8	2.1	340
**Hanks + BSA**	-1.42	79.1	2.2	296
*** Corrosion rate calculated based on the ASTM G102**				
